# Asymmetric cell division of stem cells in the lung and other systems

**DOI:** 10.3389/fcell.2014.00033

**Published:** 2014-07-31

**Authors:** Mohamed Berika, Marwa E. Elgayyar, Ahmed H. K. El-Hashash

**Affiliations:** ^1^Rehabilitation Science Department, College of Applied Medical Sciences, King Saud University, KSA and Anatomy Department, Faculty of Medicine, Mansoura UniversityMansoura, Egypt; ^2^Department of Pediatric and Neonatology, Benha Children HospitalBenha City, Egypt; ^3^Developmental Biology, Stem Cells and Regenerative Medicine Program, Keck School of Medicine and Ostrow School of Dentistry, Children's Hospital Los Angeles, University of Southern CaliforniaLos Angeles, USA

**Keywords:** stem cell, behavior, symmetric, asymmetric, cell division

## Abstract

New insights have been added to identification, behavior and cellular properties of embryonic and tissue-specific stem cells over the last few years. The modes of stem cell division, asymmetric vs. symmetric, are tightly regulated during development and regeneration. The proper choice of a stem cell to divide asymmetrically or symmetrically has great consequences for development and disease because inappropriate asymmetric division disrupts organ morphogenesis, whereas uncontrolled symmetric division induces tumorigenesis. Therefore, understanding the behavior of lung stem cells could identify innovative solutions for restoring normal morphogenesis and/or regeneration of different organs. In this concise review, we describe recent studies in our laboratory about the mode of division of lung epithelial stem cells. We also compare asymmetric cell division (ACD) in the lung stem cells with other tissues in different organisms.

## Introduction

**Figure 1:**
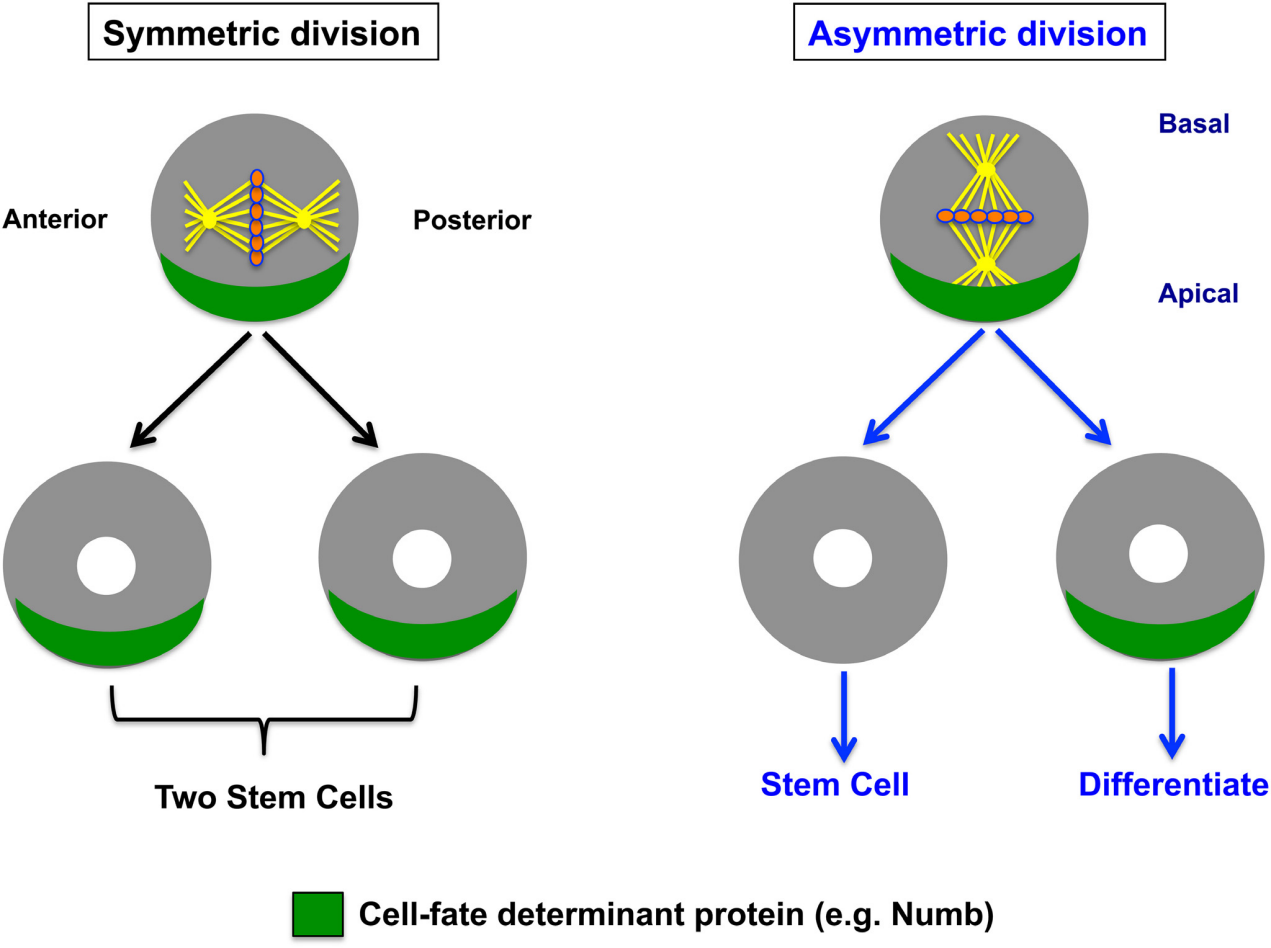
**Symmetric vs. asymmetric cell division in epithelial cells**. Schematic depiction of a polarized dividing cell show two modes of cell division. During symmetrical division, spindle orientation and determinant protein (e.g., Numb) localization are not coordinated. Determinants segregate equally, giving rise to two equal (stem) cells. During asymmetric division, spindle orientation and determinant protein (e.g., Numb) localization are coordinated, giving rise to a differentiating cell and a stem cell. Thus, the difference in Numb (green) expression levels between two daughter cells mediates asymmetric cell division, whereas lack of Numb inheritance by both daughters will allow them to execute the stem cell self-renewal program by maintaining Notch1 activity and thus allowing symmetric cell division, as we reported in distal lung epithelial stem cells (El-Hashash and Warburton, 2012).

There are two types of cell division in different organisms: symmetric and asymmetric. The major purpose of symmetric divisions is proliferation, and it therefore leads to expansion of cell populations. The symmetric division produces two identical daughter cells that acquire the same developmental fate; while the asymmetric division. On the contrary, asymmetric cell division (ACD) is a property of stem cells that gives rise to two daughter cells with different developmental fates: one daughter will differentiate along a specific lineage, whereas the other cell has the potential to renew stem cell identity and continue to divide in an asymmetric manner (Figure 1). The ability of cells to divide asymmetrically to produce two different cell types provides the cellular diversity found in every multicellular organism.

The mode of stem cell division is critical for their maintenance and expansion. Stem cells may undergo both symmetric and ACD s, instructed by diverse molecular, cellular, and environmental cues at discrete developmental stages. To distinguish these, one can look at differences in spindle orientation, or differential inheritance of cytoplasmic or membrane-bound proteins such as the cell fate determinant Numb and atypical protein kinase C (PKCζ; Huttner and Kosodo, 2005; Morrison and Kimble, 2006; El-Hashash and Warburton, 2011, 2012; El-Hashash et al., 2011). Cells divide asymmetrically in response to extrinsic or intrinsic fate determinants: extrinsically, daughter cells placed in different microenvironments adopt different fates; intrinsically, cytoplasmic cell fate determinants (e.g., Numb) are asymmetrically localized within a cell and segregate differentially into daughters that adopt different fates (reviewed by Yamashita, 2009).

The mode of cell division: symmetric vs. ACDs can support stem cell self-renewal, and is critical to maintain the proper balance between self-renewal and differentiation of stem cells (Figure 1). For instance, symmetric cell division enables stem cells to generate two daughter cells, each with properties that are indistinguishable from the mother cell, which is necessary for expanding stem cell reservoirs if these two daughters acquire stem cell fate. In addition, symmetric cell division can produce two daughters that acquire a differentiation cell fate, and therefore have less potency than the mother stem cell (Figure 1). While this may lead to rapid production of tissue “effector” cells, it can also result in potential depletion of the stem cell pool (Molofsky et al., 2004; Yamashita et al., 2010). ACD, on the other hand, is essential for balancing self-renewal and differentiation as well as correct spatial and temporal specification of cell lineages during development. In contrast to symmetric division, ACD gives rise to two daughter cells with distinct cell fates. While one daughter maintains stem cell properties, the other daughter produced by ACD loses stem cell properties and functions (Figure 1; Knoblich, 2001). Symmetric vs. ACD will be discussed in more detail in sections Asymmetry Stem Cell Divisions in Different Systems and Similarity and Significance of Asymmetric Stem Cell Division Between the Lung and Other Systems.

During development, cell divisions in the zygote produce various cell types. The ACD is the mechanism that provides the basis for many crucial developmental processes such as establishment of the body axis, cell fate determination, the maintenance of adult stem cell populations and generation of an adequate number of differentiating daughter cells. These differentiating daughter cells are vital to maintain tissue homeostasis and repair. Cell polarization is critical for asymmetric divisions. Disturbances or loss of cell polarity is often linked to enhanced stem cell self-renewal and tumorigenesis.

Asymmetric division is controlled by a combination of intrinsic and extrinsic mechanisms. Examples of intrinsic mechanisms are asymmetric localization of cell-cell junctions and/or intrinsic cell fate determinants and position within specific environment (“niche”). These are used to specify cell polarity and direct asymmetric divisions. Intrinsic mechanism involves the preferential segregation of cell fate determinants (e.g., Numb) into one of two daughter cells during mitosis (Figure 1). The successful segregation of determinants requires specialized machinery that mediates proper spindle orientation and coordinates other key events in this process. On the other hand, cell–cell communication, and thus establishment of different fates is reinforced through signaling from neighboring cells are examples of extrinsic mechanisms. For instance, interactions between daughter cells or between a daughter cell and other nearby cells in metazoans control the specification of daughter cell fate. However, multiple genes directly regulate ACDs in order to control the process of ACD itself and to determine the distinct cell fates of the two daughters.

## Asymmetric cell division in mammalian lung epithelial stem cells

In mammals, control of epithelial stem cells is essential for proper development of the lung (Warburton, 2008; Warburton et al., 2010). Lung congenital deficiencies of stem and stem cells are lethal because they affect vital processes such as gas diffusion capacity, which occurs in lung hypoplasia and bronchopulmonary dysplasia (BPD; Warburton et al., 2000, 2008, 2010; Shi et al., 2009). Providing innovative solutions to restore normal lung morphogenesis and possibly regeneration of the gas diffusion surface needs understanding how to achieve a proper balance between self-renewal/proliferation and differentiation of lung-specific stem and stem cells. During development ACD is indeed critical for balancing self-renewal and differentiation as well as correct spatial and temporal specification of cell lineages in epithelia (see Knoblich, 2001; Yamashita et al., 2010, for detailed review).

Cell polarity is identified by asymmetry in the distribution of cellular constituents within a single cell. It is crucial for various cellular processes including cell specification and migration as well as asymmetric division. Cell polarity plays a fundamental role in helping to organize and integrate complex molecular signals so that cells can make decisions concerning fate, orientation, proliferation, differentiation, and interaction (Wodarz, 2002; Nelson, 2003). Recent studies in our laboratory on lung stem cells, for instance, have shown that lung stem cells are polarized and highly mitotic with characteristic perpendicular cell divisions (detailed below). Perturbation of the polarization of lung stem cells results in loss of balance between self-renewal and differentiation of lung stem cells *in vivo* and *in culture* (El-Hashash et al., 2011).

Understanding the behavior of lung epithelial stem and stem cells may help to identify innovative solutions to restore normal lung morphogenesis. The characterization of ACD and identifying novel factors and mechanisms regulating both ACD and behavior of lung epithelial stem cells, as key mechanisms that regulate the balance between stem cell self-renewal and differentiation in the lung, can help to identify novel targets which will prevent and rescue the fatal lung disease in infancy and childhood and for lung regeneration after injury. Furthermore, identification of the molecular programs regulating the balance between the proliferation and differentiation of endogenous lung-specific stem cells is critical for developing techniques that harness the ability of these cells to regenerate diseased and damaged lungs. Despite its importance, little is known about ACD in epithelial stem cells in the lung.

Undifferentiated epithelial stem cells undergo multiple division-linked cell fate decisions (symmetric and asymmetric) in the lung, which lead to an apparently homogeneous expansion of the stem cell population (Lu et al., 2008; Rawlins, 2008). Multipotent epithelial stem cells localize within the distal lung epithelial buds/airways during embryonic development (Rawlins and Hogan, 2006; Rawlins, 2008; Rawlins et al., 2009). Recently, studies from our laboratory have indicated that ACD likely mediates the balance between lung epithelial stem cell maintenance and differentiating cell populations at distal epithelial tips. The first evidence came from our laboratory that embryonic lung distal epithelial stem cells are is polarized and highly mitotic with characteristic perpendicular cell divisions. In different mammalian epithelial cells, perpendicular cell division is strictly correlated with ACD because they undergo asymmetric division by shifting the spindle orientation from parallel to perpendicular (Lechler and Fuchs, 2005). These findings are consistent with, mouse Inscuteable (mInsc), LGN (Gpsm2), and NuMA polarity proteins, which control spindle orientation, are asymmetrically localized in mitotic distal epithelial stem cells of embryonic lungs (El-Hashash and Warburton, 2011). Interfering with the function of these polarity proteins in lung epithelial cells *in vitro* randomizes spindle orientation and changes cell fate (El-Hashash et al., 2011).

ACD is mediated by preferential segregation of intrinsic cell fate determinants (CFDs) (e.g., Numb) into one of two sibling daughter cells in *Drosophila* and mammalian epithelial cells. CFDs are asymmetrically localized in dividing cells and define the axis of polarity that will determine the orientation of the apical-basal cell division plane. This allows a rapid switch from proliferation, wherein two similar daughter cells are born, to diversification, wherein different-shaped daughter cells are generated (Betschinger and Knoblich, 2004). During interphase, Numb protein, a Notch signaling inhibitor, is expressed uniformly in the cytoplasm but is localized asymmetrically in dividing cells. Hence, Numb is segregated to only one daughter cell, enabling this cell to adopt a different fate from that of its sibling. The cell with low Numb levels maintains high Notch activity and thus has a stem cell fate whereas; the cell receiving high levels of Numb suppresses extrinsic Notch signaling and differentiates (Frise et al., 1996; Guo et al., 1996; Juven-Gershon et al., 1998; Yan et al., 2008). The cell fate determinant Numb in the embryonic lung is a key determinant of asymmetric or symmetric cell division, is highly expressed and asymmetrically distributed at the apical side of distal epithelial stem cells (El-Hashash and Warburton, 2011, 2012). Moreover, one of our recent findings is that Numb is segregated to one daughter cell in most mitotic cells (El-Hashash and Warburton, 2011). Thus, the more perpendicular/ACD is, the more likely it is to segregate Numb preferentially to one daughter cell in mitotic lung epithelial stem cells, which strongly suggest ACD in distal epithelial stem cells of embryonic lungs (El-Hashash and Warburton, 2012). Knocking down Numb in MLE15 lung epithelial cells significantly increased the number of cells expressing the stem cell markers Sox9/Id2, supporting its function as a cell fate determinant in the lung (El-Hashash and Warburton, 2012).

Epithelial cells characteristically show apical-basal polarity in many organs. They also have a distinct shape, such that only a subtle deviation in cleavage plane from the normal orientation suffices to result in an asymmetric rather than a symmetric distribution of their apical plasma membrane and adjacent adherent junctions to the daughter cells (Nelson, 2003; Kosodo et al., 2004). Using immunostaining for E-cadherin, which is a component of the apico-lateral junctional complex and lateral epithelial cell plasma membrane (Woods et al., 1997), the plasma membrane of mitotic epithelial cells show the “cadherin hole” which appears as a relatively small, unstained segment of the cell surface (Kosodo et al., 2004; El-Hashash and Warburton, 2012). Symmetric vs. asymmetric distribution of the plasma membrane to daughter cells can thus be predicted from the orientation of the cleavage plane relative to the cadherin hole in the epithelium of different organs (Kosodo et al., 2004). In our laboratory a focus on cadherin hole analyses of the lung epithelium revealed that most distal epithelial stem cells in embryonic lungs divide asymmetrically; with their cleavage, planes are predicted to bypass the cadherin hole, resulting in asymmetric distribution of the cadherin hole to the daughter cells. This provides evidence for ACD in distal epithelial stem cells of embryonic lungs (El-Hashash and Warburton, 2012).

In addition, in our laboratory we have shown that Eya1 protein phosphatase regulates cell polarity, spindle orientation and the localization of the cell fate determinant Numb, which functions as an inhibitor of Notch signaling. Thus, Eya1 promotes both perpendicular division as well as Numb asymmetric segregation to one daughter in mitotic distal lung epithelium, probably by regulating aPKCζ phosphorylation levels (El-Hashash et al., 2011). In addition, epithelial cell polarity and mitotic spindle orientation are defective after interfering with Eya1 function *in vivo* or *in vitro* (El-Hashash et al., 2011). Furthermore, we have shown that in *Eya1*^−/−^ lungs, perpendicular division is not maintained and Numb is segregated to both daughter cells in mitotic epithelial cells, leading to inactivation of Notch signaling. Moreover, they showed that genetic activation of Notch signaling, which promotes stem cell identity at the expense of differentiated cell phenotypes, could rescue the *Eya1*^−/−^ lung phenotype, which is characterized by increased epithelial differentiation but reduced branching and loss of epithelial stem cells.

In our laboratory, we have indicated that Eya1 protein phosphatase controls the balance between self-renewal and differentiation of distal lung epithelial stem cells by regulating ACD, which is critical for the long-term maintenance of tissue self-renewal during development and in diseases. For instance, congenital lung hypoplasia and bronchopulmonary dysplasia (BPD), wherein a significant deficiency of stem cells probably occurs, are common features of human prematurity and/or lung injury and are thus major public health problems in human infancy. Proper balance between self-renewal and differentiation of lung-specific stem cells, which is mediated by ACD, is absolutely required for normal lung morphogenesis and regeneration. In order to generate a sufficiently large gas diffusion surface to sustain life, regulated outgrowth and branching of the epithelial tubes is essential. Defective differentiation and postnatal respiratory distress are direct results of developmental defects in this smooth progression (Warburton et al., 2008, 2010).

To summarize, our laboratory provides several lines of evidence suggesting that ACD s are common in embryonic distal lung epithelial stem cell populations. For instance, the cleavage plane orientations are predicted to bypass the cadherin hole, resulting in asymmetric distribution of the cadherin hole to the daughter cells in most distal epithelial stem cells (El-Hashash and Warburton, 2012). In addition, our report that most of the distal epithelial cells have apically localized Par, LGN, NuMA, and mInsc polarity proteins, with mitotic spindles aligned perpendicular to the basement membrane and a characteristic asymmetric segregation/inheritance of Numb (El-Hashash and Warburton, 2011), provides further evidence that they are dividing asymmetrically. There is a strict correlation between ACD and the apical localization of polarity proteins Par/LGN/NuMA/mInsc, which control spindle orientation in mammalian epithelial mitotic cells (Lechler and Fuchs, 2005), perpendicular alignment of mitotic spindles, and asymmetric Numb segregation in different *Drosophila* and mammalian epithelial cell types (Cayouette and Raff, 2002, 2003; Haydar et al., 2003; Noctor et al., 2004; Lechler and Fuchs, 2005). Further investigations are needed to determine asymmetric vs. symmetric division in embryonic lung distal epithelial stem cells.

## Asymmetry stem cell divisions in different systems

ACD has been reported in different tissue types in the animal kingdom. Factors and molecular mechanisms that act to specify cell fate and orient mitotic spindles during ACD are still not fully understood. Activation of the Notch signaling pathway and/or asymmetric segregation of the Notch inhibitor Numb are common mechanisms of ACD across a number of stem cell systems. In this section, examples of ACD that are dependent on Notch signaling activity and occur in non-mammalian or mammalian systems will be reviewed.

### Intestinal stem cells (ISCs) of drosophila

ISC is a well-studied model system for ACD. It is demonstrated that ISCs act to maintain the intestinal epithelium. ISCs reside within clusters of 2–3 basally located diploid cells, which are interspersed between polyploid enterocytes along the intestinal basement membrane. In addition, they generate the hormone-producing enteroendocrine cells and polyploid enterocytes (Micchelli and Perrimon, 2006; Ohlstein and Spradling, 2006).

Ohlstein and Spradling (2007) have shown that the Notch signaling activity is critical for mediating asymmetric division of ISCs in the midgut of adult flies. They also demonstrated that Notch target genes in the daughter enteroblasts were activated by ISCs signal through Delta. They noticed that Notch signaling is activated in the daughter enteroblast exclusively. In addition, only the ISC in a direct contact with the basement membrane stained positive for the Notch ligand Delta. Furthermore, Notch positivity was detected in all cells in the stem cell containing clusters (Ohlstein and Spradling, 2007). The molecular mechanism that facilitates the asymmetric division by blocking of Notch signaling activity within the ISC is not investigated thoroughly. However, the analysis of mitotic spindle orientation in dividing ISCs by many studies demonstrated that these stem cells divide non-randomly, so that the daughter ISC that remains in contact with the basement membrane remains an ISC, whereas the daughter cell that is displaced away undergo differentiation to form an enteroblast (Toledano and Jones, 2009). Orientations of the mitotic spindle and the mechanism(s) controlling this process in ISCs have not been characterized.

### Hematopoietic stem cells (HSCs)

Several studies suggest the importance of Notch signaling in regulating the fate of HSCs by blocking differentiation exactly as in Drosophila ISCs (Duncan et al., 2005; Wu et al., 2007). These studies showed that a Notch-responsive GFP reporter in transgenic mice could be used to enrich for hematopoietic stem cells. This transgenic Notch reporter strain, shows GFP^+^ cells contained approximately 40–60% HSCs, whereas in differentiating precursors expression of GFP is significantly reduced (Duncan et al., 2005; Wu et al., 2007). In addition, this transgenic Notch reporter strain and real time imaging in order to visualize hematopoietic precursor cell divisions growing in culture. Furthermore, Duncan et al. (2005) and Wu et al. (2007) highlighted the importance of both extrinsic cues and intracellular factors in the control of hematopoietic precursor cell divisions. They used these research tools to show that different types of oncogenic chromosomal translocations such as BCR-ABL can affect on either the pattern of cell divisions or cell proliferation and survival of hematopoietic stem cells. However, further investigation is needed to understand the molecular mechanisms involved in the regulation of ACD by these factors. Important questions are still waiting for answers such as are the niche and the surrounding different cell types, including vascular endothelial cells, osteoblasts and stromal reticular cells also influence the orientation pattern of mitotic spindles and ACDs of HSCs? Furthermore, what is the relative importance of the ability of hematopoietic stem cells to divide asymmetrically to the blood homeostasis?

### Muscle stem cells

Adjacent to the mature myofibers, satellite cells reside beneath the basement membrane and are effectively acting as muscle stem cells. Satellite cells are normally quiescent but can be induced to enter the cell cycle upon injury. They are essential to maintain production of myoblasts during postnatal development and during muscle repair after injury. Different daughter cells in dividing muscle-lineage cells during muscle growth and regeneration show asymmetric segregation of older (immortal) and younger DNA strands (Cairns, 1975; Shinin et al., 2006; Conboy et al., 2007). Other studies *in culture* and *in vivo* have shown induced expression of several differentiation genes and differential localization of cell fate proteins; including Numb, within daughter cells. Those studies provide evidence that muscle stem cells can undergo ACDs (Conboy and Rando, 2002; Shinin et al., 2006; Kuang et al., 2007). The relative importance of ACD in muscle regeneration needs further investigation. In addition, the molecular mechanisms that control muscle satellite cell division in response to various environmental cues and factors involved in the determination of daughter cell fates yet have to be established.

### Epidermal stem cells in mammals

The skin is the largest organ of the body. It provides a protective barrier against the outside world. Damage of that barrier is potentially lethal and must be repaired rapidly and efficiently. The cells of the basal cell layer of the epidermis proliferate periodically to replicate themselves and to produce the supra-basal layers, which move outward and eventually die. The molecular factors responsible for ACDs are conserved throughout evolution. Several studies on skin cells growing in culture and on mouse embryos *in vivo* have shown evidences of both symmetric and ACD in epidermal stem cells in mammals. In addition, there is evidence of ACDs within the basal layer of the esophageal epithelium (Seery and Watt, 2000). Furthermore, several studies have shown that stem cells in the basal layer of the epidermis replicate symmetrically to produce more stem cells and asymmetrically, to generate the stratified epithelium of the epidermis (Smart, 1970; Lechler and Fuchs, 2005). Using ACD, the epidermal stem cells produce two cells, one is a proliferative cell which remains in the basal layer in contact with the baso-lateral membrane and another cell which detaches from the basal membrane to become in the supra-basal' layer and displace apically toward the surface of the skin (Smart, 1970; Lechler and Fuchs, 2005). The supra-basal cells stop dividing and form the barrier layer of the skin by entering a differentiation program (Fuchs and Raghavan, 2002).

**Figure 2:**
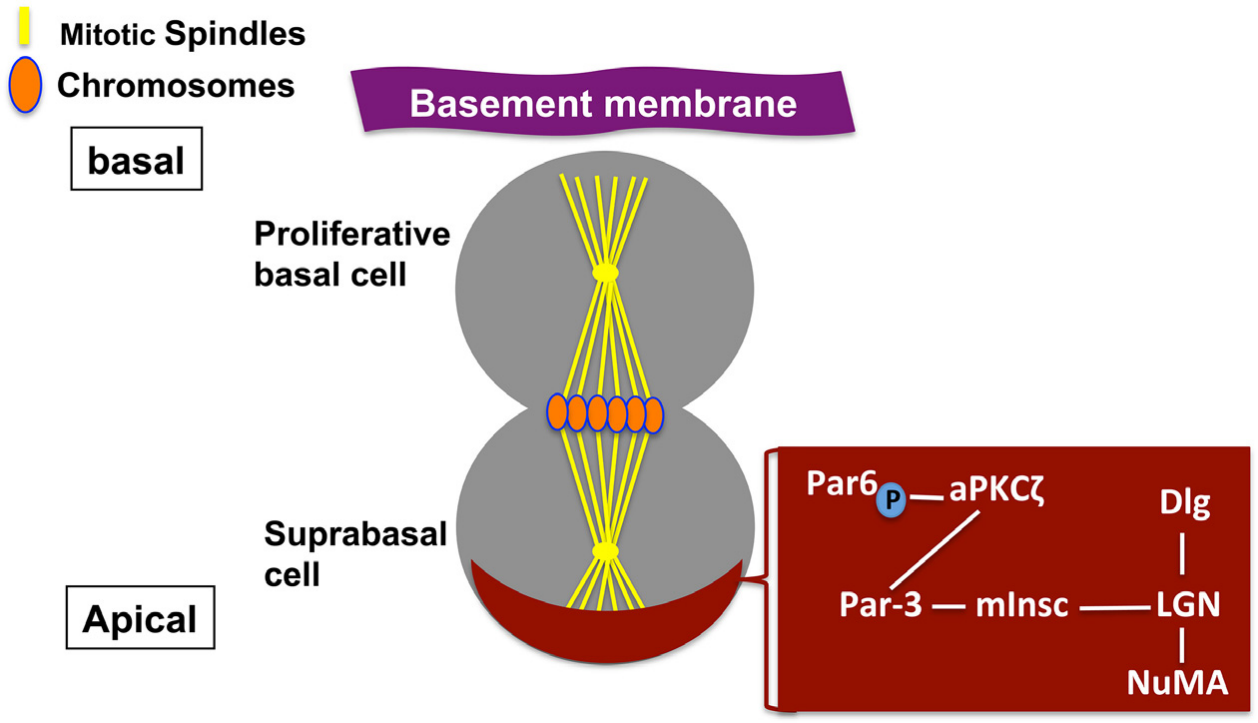
**Asymmetric cell division in mammalian epithelia**. Schematic depiction of a polarized mammalian mother cell during mitosis (anaphase). Apical protein complexes are shown as a brown crescent. These apical protein complexes are important for both polarity establishment and spindle orientation in mammalian cells and are shown in a brown box, and described in the text.

Lechler and Fuchs (2005), showed that the mitotic spindles are perpendicular to the basement membrane in asymmetrically dividing cells during stratification of the skin. In addition, they showed that basal stem cells use integrins and cadherins in order to achieve physical attachment to the underlying basement membrane. These adhesion molecules are important for spindle alignment. Furthermore, they reported a concentration of both growth factor receptors and integrins at the base of the cell that could influence stem cell stem cell maintenance and behavior. Lechler and Fuchs (2005) studied the perpendicular cell divisions and demonstrated that they act as a natural mechanism for the unequal partitioning of the signaling molecules derived from the basement membrane into the two daughters. In mammalian epithelial stem cells, the apical complexes of polarity proteins are formed opposite the basement membrane and are segregated into the supra-basal cell that to be ready for differentiation (Figure 2). In asymmetric divisions, the mitotic cells with perpendicular spindles have an apical crescent of cortical LGN, which is considered as the mammalian Pins ortholog. LGN, in turn, binds both mouse Inscuteable (mInsc) and Par3 at the apical cortex of the basal cells. Another polarity protein is the atypical PKC (aPKCζ), which localizes to the apical cortex of the basal cells (Lechler and Fuchs, 2005; Figure 2). Furthermore, LGN binds the Mud ortholog NuMA, which tethers spindles at the poles (Du et al., 2001). Integrins and cadherins are essential for the apical localization of aPKCζ, the Par3-LGN-Inscuteable complex and NuMA-dynactin to align the spindle in basal stem cells (Lechler and Fuchs, 2005). However, the primary function of the apical polarity proteins in mammalian epithelial cells is the determination of mitotic spindle positioning and establishment of apico-basal polarity, rather than the specification of stem cell fate (see Macara, 2004a,b; Lechler and Fuchs, 2005; Suzuki and Ohno, 2006; Shin et al., 2007; Knoblich, 2010; Yamashita et al., 2010 for detailed review).

Another important factor of ACDs in mammalian epidermal stem cells is the transcription factor p63, which stimulates epidermal proliferation (Mills et al., 1999; Yang et al., 1999). Basal cells only divide symmetrically in the absence of p63. This suggested that p63 is required for stratification (Lechler and Fuchs, 2005; Senoo et al., 2007).

Similar to *Drosophila* neuroblasts, the asymmetric activation of the Notch pathway mechanism was used in order to ensure an asymmetric outcome of mammalian epithelial stem cell divisions. In addition, it was found that supra-basal cells utilize the Notch intracellular domain (NICD) to promote differentiation (Blanpain et al., 2006). Furthermore, Smith et al. (2007) have shown that Notch signaling is inhibited by the cell fate determinant Numb that localizes primarily to the baso-lateral cortex as a result of aPKCζ-mediated phosphorylation. This results in its exclusion from the apical pole in cultured mammalian epithelial cells. However, other molecular mechanisms and specific cell fate determinants that regulate ACDs, cell fate and behavior of mammalian epidermal stem cell during developmental and adult stages still need more investigation.

### Mammalian neural stem cells

Many authors have demonstrated that symmetric and ACDs occur at different developmental stages of neural stem cells in mammals. ACDs occur in the neuroepithelium of the vertebrate retina and ventricular zone of the cerebral cortex in mammals (Gonczy, 2008; Neumüller and Knoblich, 2009; Yamashita et al., 2010). Interestingly, during early developmental stages symmetric cell divisions also occur and are primarily reported probably to increase the neural stem cell population, while ACDs took place later on in order to generate differentiating neurons.

In the vertebrate nervous system, spindle orientation is regulated by several factors such as Gα-binding protein LGN, mouse Inscuteable (mInsc) and other factors have conserved roles similar to other systems. Furthermore, Notch signaling components are influence cell fate decisions in this system in vertebrate nervous system (Chenn and McConnell, 1995; Zhong et al., 1996, 1997; Petersen et al., 2004). Other studies suggest that notch signaling is inhibited by the cell fate determinant Numb. In addition, Numblike may influence cell fate by mechanisms other than inhibition of Notch signaling activity (Rasin et al., 2007; Zhou et al., 2007).

Knoblich (2001, 2010) has reviewed the role of the cell fate determinant Numb as a key determinant of asymmetric vs. symmetric cell division. Both ACD and Numb expression as well as function during development have been comparatively well studied in the mammalian nervous system and *Drosophila* (Betschinger and Knoblich, 2004), but they are not characterized in the lung.

During the development of vertebrate nervous system the correlation between mitotic spindle orientation and cell fate determination is not yet clear. In many other systems the spindle positioning is an indicator of whether cell divides symmetrically or asymmetrically (Sanada and Tsai, 2005; Zigman et al., 2005; Morin et al., 2007; Konno et al., 2008). For example, disruption of the activity of Gα-binding protein LGN, a key regulator of mitotic spindle orientation, in the spinal cord neuroepithelium leads to randomization of spindle orientation without disrupting daughter cell fate (Morin et al., 2007). On the other hand, reduction of another essential regulator of spindle orientation, mouse Inscuteable (mInsc) in the retina leads to disruption of mitotic spindle orientation and an increase in stem cells and neuronal defects (Zigman et al., 2005; see Figure 2).

Furthermore, a recent study by Forostyak and colleagues (Forostyak et al., 2013) has demonstrated the importance of Ca^2+^, its homeostasis and signaling potential in human embryonic stem cells (hESCs) differentiated to a neuronal phenotype. In this study, they have identified the molecular cascades of [Ca^2+^]_*i*_ homeostasis and Ca^2+^ signaling in correlation with hESC differentiation into a neuronal phenotype. They have also analyzed changes in cytoplasmic Ca^2+^ concentration ([Ca^2+^]_*i*_) evoked by high K^+^, adenosine-5′ triphosphate (ATP), glutamate, γ-aminobutyric acid (GABA), and caffeine in correlation with the expression of various neuronal markers in different passages (P6 through P10) during the course of hESC differentiation. This study has shown that only differentiated neural precursors (NPs) from P7 exhibited significant and specific [Ca^2+^]_*i*_ responses to various stimuli. Thus, almost 31% of neuronal-like P7 NPs exhibited spontaneous [Ca^2+^]_*i*_ oscillations. In addition, P7 NPs express L- and P/Q-type Ca^2+^ channels, P2X_2_, P2X_3_, P2X_7_, and P2Y purinoreceptors, glutamate receptors, and ryanodine (RyR1 and RyR3) receptors (Forostyak et al., 2013). Moreover, they have provided evidences that the ATP- and glutamate-induced [Ca^2+^]_*i*_ responses were concentration-dependent, and that responses to ATP were observed in the presence or in the absence of extracellular Ca^2+^. These data by Forostyak et al. (2013) strongly suggest that with time in culture, hESCs cells attain a transient period of operative Ca^2+^ signaling that is predictive of their ability to act as stem elements.

## Similarity and significance of asymmetric stem cell division between the lung and other systems

Studies in our laboratory have shown a similarity in the mode of stem cell division between the lung and other systems. For instance, similar to stem cells of different tissues (Lechler and Fuchs, 2005; Yamashita et al., 2010), lung distal epithelial stem cells are polarized with perpendicular rather than parallel divisions (El-Hashash and Warburton, 2011). In addition, we found that asymmetric segregation and inheritance of Numb may be a common mode of ACD control in lung stem cells (El-Hashash et al., 2011; El-Hashash and Warburton, 2012), similar to neural stem cells and satellite muscle cells (reviewed in Morrison and Kimble, 2006). Another aspect of similarity is the apical localization of polarity proteins Par/LGN/NuMA/mInsc, which control spindle orientation in mammalian epithelial mitotic cells (Lechler and Fuchs, 2005). We reported that most distal lung epithelial stem cells have apically localized Par, LGN, NuMA, and mInsc polarity proteins, with mitotic spindles aligned perpendicular to the basement membrane. Both polarized localization of Par, LGN, NuMA, and mInsc proteins, and perpendicular alignment of mitotic spindles are strictly correlated with ACD in Drosophila and mammalian epithelial cell types (Cayouette and Raff, 2002, 2003; Haydar et al., 2003; Noctor et al., 2004; Lechler and Fuchs, 2005).

ACD mediates the balance between stem cell self-renewal and differentiation in different systems (Yamashita et al., 2010). This ACD-mediated balance is critical for the long-term maintenance of tissue self-renewal during development and in diseases in different organs, including the lung. For example, bronchopulmonary dysplasia (BPD) and congenital lung hypoplasia, wherein a significant deficiency of stem cells probably occurs, are common features of human prematurity and/or lung injury and are thus major public health problems in human infancy. Therefore, the proper balance between self-renewal and differentiation of lung-specific stem cells that is mediated by ACD, is most likely required for normal lung development, repair and regeneration. Indeed, tightly controlled outgrowth and branching of the epithelial tubes in the lung generate a sufficiently large gas diffusion surface to sustain life. Developmental defects in this smooth progression may, therefore, lead to defective differentiation and postnatal respiratory distress (Warburton et al., 2008, 2010). Our data that show similarities of asymmetric stem cell division between the lung and other systems could identify innovative solutions to restoring normal lung morphogenesis.

## Concluding remarks and future directions

Much insight into different mechanisms that are necessary to generate cellular diversity and maintain stem cells have been demonstrated by several recent studies that focused on ACDs across various species and in multiple stem cell systems. Studies using invertebrate model systems such as *Drosophila* have identified the importance of several extrinsic signals and intrinsic factors in stem cell division pattern and provided paradigms for how both these signals and factors act to specify asymmetric divisions. Many recent studies provide evidence that similar mechanisms are used in vertebrates. However, proper characterization of stem cells *in vivo*, advanced isolation of pure populations of stem cells, and improvements in real time imaging are still needed to facilitate studies that aim at the identification and determination of the mechanisms regulating ACDs in more complex mammalian stem cell systems, including humans.

Proper balance of the number of stem and stem cells is also essential during organ development, repair and regeneration. Many recent studies on the mechanisms regulating asymmetric stem cell divisions have shown the importance of the balance of the number of stem cells. The correct balance and tight control of the number of stem cells by asymmetric divisions are not only important during the establishment and maintenance of tissues, but also critical during tissue repair and regeneration, This is because an increase in the number of symmetric divisions may be required temporarily to increase the number of stem cells during tissue repair and regeneration. It is important to consider that several factors can act to hinder or even prevent stem cell from switching from symmetric back to asymmetric mode of cell divisions. For instance, chronic injury or inflammation of a tissue might compromise the ability of stem cells to respond appropriately to repair damaged tissues. It also may cause failure of stem cells to switch from symmetric to asymmetric mode of divisions. Failure of the proper regulation of tissue repair could eventually lead to the selection of stem cells that are resistant to normal growth control signals, which is a hallmark of cancer cells. Therefore, understanding signaling mechanisms that regulate ACD in all types of stem cells is critical for developing techniques that harness the ability of these cells to regenerate diseased and damaged organs. In addition, understanding these mechanisms will most likely help to design potent strategies to inhibit cancer initiation in different cell types and may also identify new targets for anti-cancer therapies. Moreover, identification of the molecular mechanisms and factors regulating the behavior of adult stem cells will be essential for both the expansion and maintenance of stem cells in culture, while maintaining their differentiation potential. This will also direct the differentiation of stem cells into different specialized cell types ready for use in regenerative medicine.

Our recent studies on the characterization of ACD, and future studies on the identification of new factors and mechanisms that regulate ACD in lung epithelial stem cells, which is a key mechanism regulating the balance between lung stem cell self-renewal and differentiation, may help to identify novel targets for the prevention and rescue therapy of fatal lung disease in infancy and childhood and for lung regeneration after injury. In addition, future studies on the identification of molecular programs that control the balance between self-renewal and differentiation of endogenous lung-specific stem cells will be crucial for developing techniques that harness the ability of these cells to regenerate diseased and damaged lungs.

### Conflict of interest statement

The authors declare that the research was conducted in the absence of any commercial or financial relationships that could be construed as a potential conflict of interest.
